# Persistence of *Wolbachia* DNA in *Dirofilaria immitis-*infected canines after doxycycline-only treatment in Rio de Janeiro State, Brazil

**DOI:** 10.1186/s13071-026-07621-9

**Published:** 2026-09-01

**Authors:** Doug Carithers, Bruno Alberigi, Fabio Scott, Flavya Mendes-de-Almeida, Debora Borges, Norma Labarthe, Yara Brandão, Regiane S. Cunha, Edneia A. de Souza Ramos, Marcelo Molento, Stephen Yoon, John McCall, John Harrington

**Affiliations:** 1 Boehringer Ingelheim, Duluth, GA USA; 2 Programa de Pós-Graduação em Ciências Veterinárias, Universidade Federal Rural do Rio de Janeiro, Seropédica, RJ Brazil; 3 Laboratório de Quimioterapia Experimental em Parasitologia Veterinária, Universidade Federal Rural do Rio de Janeiro, Seropédica, RJ Brazil; 4 Departamento de Patologia e Clínica Veterinária, Universidade Federal Fluminense, Niterói, RJ Brazil; 5 Laboratório de Parasitologia Clínica Veterinária, Universidade Federal do Paraná, Curitiba, PR Brazil; 6 Departamento de Patologia Básica, Universidade Federal do Paraná, Curitiba, PR Brazil; 7 Boehringer Ingelheim, Athens, GA USA; 8 TRS Labs Inc.,, Athens, GA USA

## Abstract

**Background:**

*Wolbachia* are obligatory bacterial endosymbionts of *Dirofilaria immitis* heartworms, necessary for worm development, reproductive capability, and viability. We quantitatively examined microfilariae and *Wolbachia* in doxycycline-treated, heartworm-positive dogs.

**Methods:**

Microfilaremic heartworm-positive dogs were identified in municipalities within the target areas of Rio de Janeiro and enrolled in this study. Dog owners agreed to allow blood draws from their dogs on day 0 and days 60 and 90 (± 5 days), and to administer doxycycline 10 mg/kg BID for 28 days, with weekly phone follow-up to help ensure compliance. Modified Knott’s tests were performed on each experimental day; a portion of each sample was retained and frozen at − 20 °C, and the remaining quantities were submitted for digital PCR (dPCR) of microfilarial and *Wolbachia* burdens. None of the dogs enrolled in the study received any macrocyclic lactone during the study.

**Results:**

Thirty-one microfilaremic dogs completed the study, with 27 remaining microfilaremic throughout. *Wolbachia* were cleared from 25 of the dogs by day 90, demonstrating that the doxycycline dose was fully effective in those cases. Interestingly, 6 dogs remained dPCR positive on the quantitative assessment, and the proportions of *Wolbachia*/*D. immitis* on days 60 and 90 were significantly (*p *< .0001) lower than on day 0. Results of the comparisons of proportions for day 60 vs. day 90 were not statistically significant (*p* = 0.3127).

**Conclusions:**

This study demonstrates that *Wolbachia* DNA may persist in some dogs for 90 days after doxycycline-only therapy. These data may reflect the lack of macrocyclic lactone use or higher adult burdens in some dogs; however, it is not possible to confidently conclude these factors entirely underpin our observations. We suggest that further studies should investigate whether this is related to lack of efficacy of doxycycline in certain *Wolbachia* subpopulations or to other factors.

**Graphical Abstract:**

**Figure:**
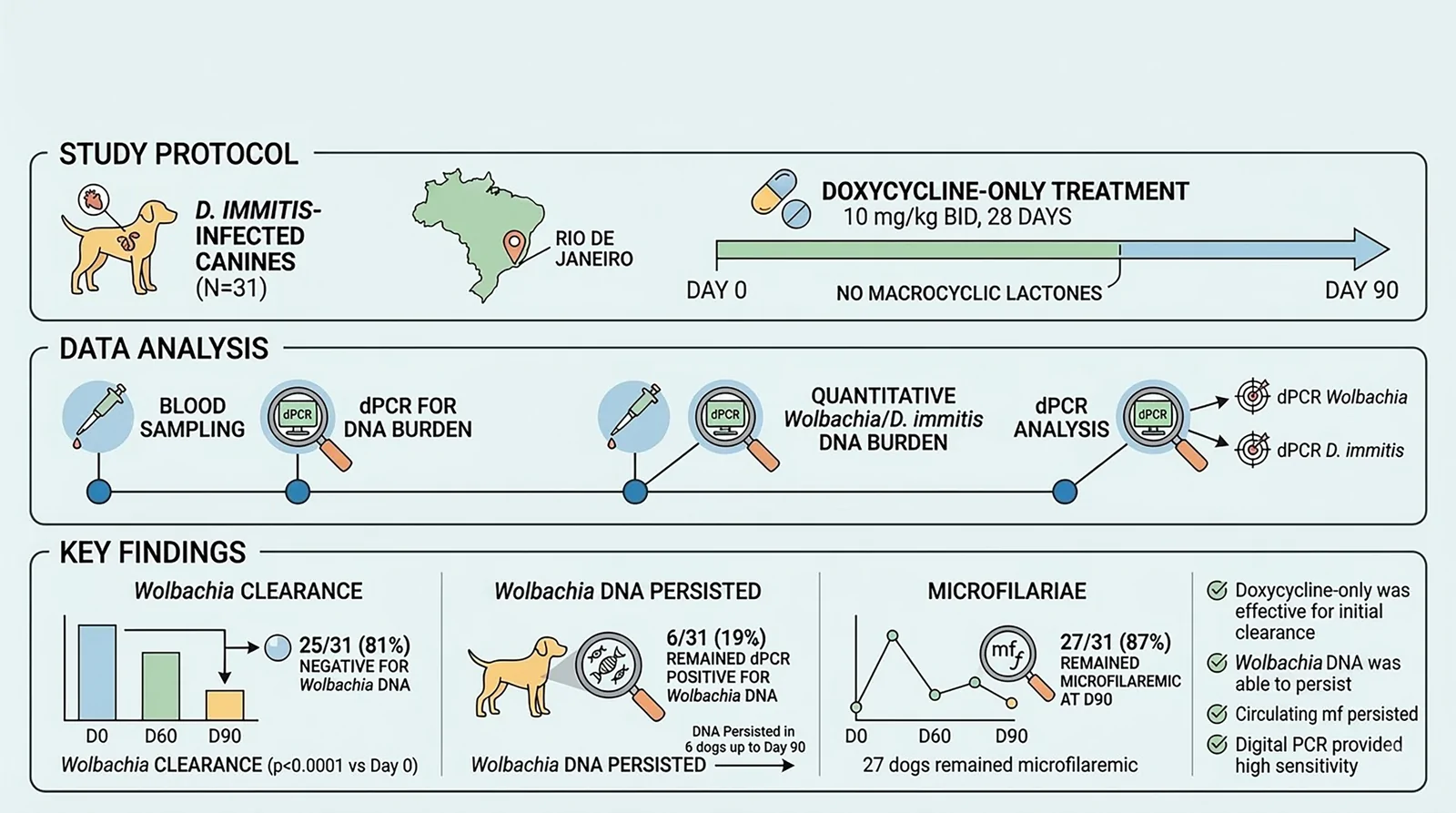

**Supplementary Information:**

The online version contains supplementary material available at https://doi.org/10.1186/s13071-026-07621-9.

## Background

*Wolbachia* are gram-negative endosymbionts of some arthropods and are transmitted vertically and horizontally, but as endosymbionts of most filarial nematodes, are transmitted only vertically. They are obligatory bacterial endosymbionts of *Dirofilaria immitis* (the canine heartworm) and necessary for worm development, reproductive capability, and viability [1–3]. Adult female heartworms can release over 11,000 microfilariae per day, and each microfilaria only receives *Wolbachia* endosymbionts from its mother [4]. *Wolbachia* play an essential role in the metabolism of filarial nematodes, producing enzymes required for the biosynthesis of folate, purines, pyrimidines, and heme [2, 5]. While molting in the vector and in vitro (L3–L4) appear to be independent of the presence of *Wolbachia* [6], impairment of further development in the host likely occurs post L4 development and may involve germline stem cell dysregulation as seen in the related filarial nematode *Brugia malayi* [7] and host immune factors as demonstrated in another filarial nematode *Litomosoides sigmodontis* [8]. Indeed, it has been shown that *Wolbachia* may help worms evade the canine’s immune response [9]. Due to this intricate bacterial–filarial interaction, the use of doxycycline has proven efficacious for reducing the *Wolbachia* burden in *D. immitis,* subsequently reducing its fitness [10–19].

In competent mosquito species, ingested doxycycline-treated microfilariae of *D. immitis* can still migrate into the Malpighian tubules of the mosquito [17, 18]. There, the microfilariae develop into first-, second-, and third-stage larvae (L3), ultimately exiting the Malpighian tubules and migrating through the hemocoel to the mosquito's head and mouthparts, where they become infective L3. Doxycycline-treated infective L3 still exit the mouthparts of the mosquito onto the surface of the skin and infect the vertebrate host during the next blood meal of the mosquito vector. Yet they do not develop normally in the vertebrate host and fail to reach the adult stage [16–18]. The specific mechanisms by which larvae fail to develop into adults remain to be elucidated.

Despite advancements in heartworm therapy and our improved understanding of doxycycline's role in eliminating or reducing *Wolbachia* burden in *D. immitis*, the impact of doxycycline treatment has been assessed quantitatively only to a limited extent. In this study, we quantitatively examined microfilariae and *Wolbachia* after doxycycline-only treatment in heartworm-naturally infected dogs.

## Methods

### Study area

**Fig. 1:**
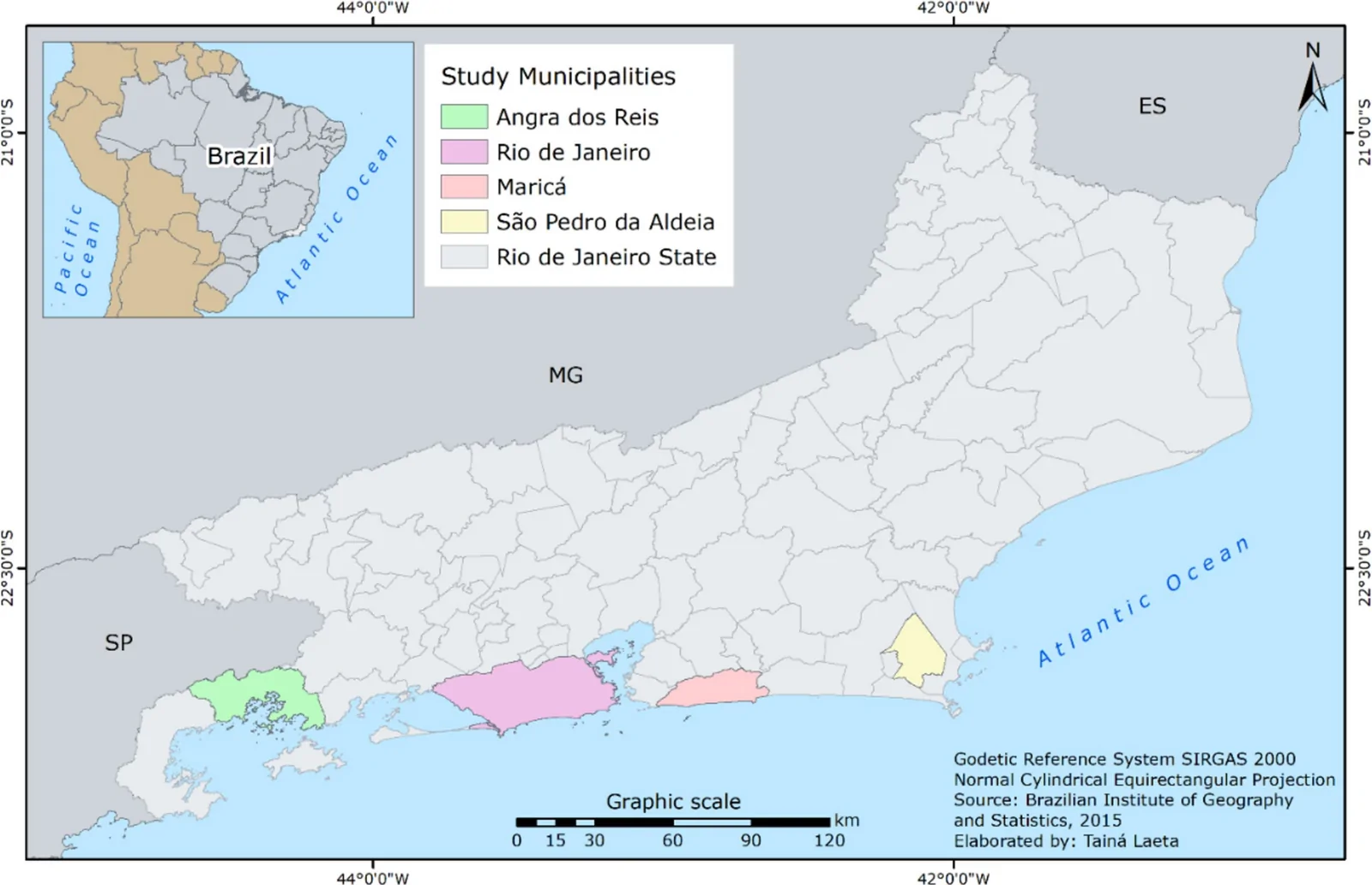
Map showing the municipalities in the state of Rio de Janeiro, Brazil, from which dogs infected with *Dirofilaria immitis* were sampled

Study recruitment was targeted across four geographic locations known to be endemic for heartworm infection in the State of Rio de Janeiro: Angra dos Reis, Rio de Janeiro, Maricá, and São Pedro da Aldeia (Fig. 1). These sites were selected to represent topographically diverse habitats and varying population densities, providing a more comprehensive sampling of dogs in the area. Angra dos Reis (Angra) is a coastal municipality of 816km^2^ in the Southern area of the state. Its lowlands are bordered by the ocean and by hills covered by Atlantic Forest. The rainfall index is high (˜130 days/year), relative humidity is high (81.5%), and the mean temperature is warm (20–27 °C). Tourism is very active in the area once the seawater is warm, and there are 365 islands and many waterfalls. Rio de Janeiro (Rio) is coastal, with a high human population density (5174/km^2^), a large urban forest (˜40 km^2^), and ample standing water, which allows mosquito breeding. The rainfall index is high (˜121 days/year), relative humidity is high (79%), and the mean temperature is warm (19–27 °C). Maricá is a 361 km^2^ coastal area characterized by standing water and large sandbars. The rainfall index is lower (˜94 days/year), the relative humidity is high (79%), and the mean temperature ranges from 19 °C to 28 °C. São Pedro da Aldeia (São Pedro) is a 333 km^2^ municipality located on a large lagoon with salt marshes and sandbars, known for being a breeding site for heartworm vectors. It is located in the extreme eastern section of the state, with a low rainfall index (< 100 days/year), a mean temperature ranging from 19 °C to 29 °C, and a mean relative humidity of 75%.

### Patient selection

Signed informed consent for participation of the dogs in the study was obtained from all dog owners before screening for inclusion of the dogs in the study (Appendix 1). The study was approved by the Institutional Animal Care and Use Committee of the Veterinary Institute of the UFRRJ (CEUA/IV/UFRRJ), approval number 2886031121.

The target for inclusion in the study was a minimum of 30 microfilaremic *D. immitis*-infected dogs who were not receiving any macrocyclic lactone formulation, with or without a history of doxycycline. Any dogs receiving macrocyclic lactones were excluded to avoid synergism or bias in the results. Any dogs enrolled but later found not able to tolerate doxycycline (e.g., gastrointestinal upset that did not respond to symptomatic treatment within 48 h) were removed from the study.

The inclusion criteria were as follows: (1) healthy dogs, other than being heartworm-positive; (2) weighing at least 15 kg (live weight) to comply with Brazilian ethical requirements; (3) presenting a body condition score ranging from ideal to overweight; (4) harboring at least three microfilariae (mf) at a low power field on a fresh blood sample; (5) presenting *D. immitis* antigenemia; and (6) pet owners willing to have their dog(s) participate in a 90-day study, to administer doxycycline BID for 28 days and to return with the dog for blood sampling on days + 60 (± 5 days), and + 90 (± 5 days).

After the dog owners provided formal consent for their dogs to participate in the study, the dogs were sampled for *D. immitis* diagnosis. About 16 ml of blood was drawn from each dog using a closed system (vacuum 4 EDTA 4 ml tubes with 21- or 23-gauge butterflies). All tubes were uniquely labeled. Immediately after sampling, 0.05 ml of blood from each dog was examined for microfilariae. The microfilariae-positive samples were counted using Knott’s modified test. When the microfilariae concentration exceeded 300 microfilariae/mL, a second infection test was performed with an antigen ELISA-based platform confirming *D. immitis* antigenemia (SNAP Test 4Dx Plus, IDEXX Laboratory, USA). All microfilariae were assumed to be *D. immitis,* as there are no reports of *D. repens* in Rio de Janeiro state. When a dog tested positive for both microfilaremia and antigenemia, it was weighed, given a physical exam, and other information (such as home address) was recorded (Appendix 2).

The owners received doxycycline pills (Doxitrat^®^, Agener União, São Paulo, Brazil) to be administered orally at 10 mg/kg BID for 28 consecutive days, starting on day 0. All owners were instructed to administer the medication and to observe whether the animal showed discomfort or vomiting. If any adverse reaction was observed, the local team was instructed to call the Rio de Janeiro team's emergency phone number. Investigators called the dog owners every 7 days to ensure treatments were administered appropriately.

Dogs were sampled using the same procedures on days 0, 60, and 90 after the first dose of doxycycline. Day 30 was not included, because the impact of doxycycline on *Wolbachia* and the consequent impact on microfilarial concentration has been reported to be a slow process [19, 20], and to avoid owners’ noncompliance. A modified version of Knott’s test [21] was performed to identify and count the microfilariae of each sample. Knott’s test was performed using 1 ml of blood in 9 ml of 2% formalin, centrifuged at 400 g for 5 min. The supernatant was discarded, and 100 μl of the sediment was diluted in 100 μl of methylene blue (1:1000). A 20 μl subsample was examined under the microscope, and microfilariae were counted. The final microfilariae count/ml blood was the subsample count multiplied by 10.

Once all fresh blood tests had been performed, the four remaining tubes from each dog were frozen at -20 °C. Two frozen blood sample tubes from each dog were shipped to the Laboratory of Veterinary Clinical Parasitology at Universidade Federal do Paraná (UFPR) in order to arrive no more than 6 days after sampling. The other two tubes were retained and frozen at Universidade Federal Rural do Rio de Janeiro. 96 samples were shipped to the Paraná Team.

### DNA extraction

Blood samples were collected from 31 antigen-positive dogs which were microfilaremic on day 0. DNA extraction was performed using the ReliaPrep Blood gDNA Miniprep System kit (Promega, Wisconsin, USA) according to the manufacturer's instructions. The concentration and purity of the DNA were assessed by spectrophotometry using a NanoDrop One from Thermo Scientific (Massachusetts, USA). The DNA was kept at -20 °C until used.

### Digital polymerase chain reaction (dPCR)

Species-specific primers and IDT probes (Iowa, USA) [22] were used for dPCR (Table 1). The samples were standardized by first testing the primers in a conventional PCR. Subsequently, variations in probe and primer concentrations, initial DNA amount, and temperature in the dPCR reactions were tested. After standardization, duplex dPCR reactions were performed with a final volume of 40 μl. The reaction was set up under the following conditions: 20 ng of DNA; 1X Probe PCR Master Mix from Qiagen (Hilden, Germany), 0.8 μM from each primer for *Wolbachia*, 0.4 μM probe for *Wolbachia*, 0.6 μM of each primer for *D. immitis*, 0.3 μM probe for *D. immitis*, 0.2 IU of EcoRI (Thermo Scientific, Massachusetts, USA). The reaction volume was pipetted into the well of Qiagen's Nanoplate 26 K 24-well plate (Hilden, Germany). The reaction was performed on Qiagen's QIAcuity One equipment (Hilden, Germany) under the following temperature conditions: 1 cycle at 95 °C for 2 min, followed by 55 cycles at 95 °C for 15 s, 58 °C for 15 s, and 72 °C for 15 s. The exposure time was 500 ms, and the gain was 6. A negative control without DNA (NTC) was inserted in all plates. The fluorescence threshold line was manually set for all samples, following the equipment's established methodology.

**Table 1:** Primers and probes used to run dPCR for *Dirofilaria immitis* and *Wolbachia* in blood samples from dogs in Rio de Janeiro, Brazil

	*D. immitis* (*Cox-1* gene)	*Wolbachia* (*Ftsz* gene)
Forward 5’	CATCCTGAGGTTTATGTTATTATTTT	AAGCCATTTGGCTTCGAAGGTG
Reverse 3’	CTGTATACATATGATGACCCCA	AAACAAGTTTTGATTTGGAATAACAAT
Probe	CGGTGTTTGGGATTGTTAGTG	CGTATTGCAGAGCTCGGATTA
Modification of 5' probe	HEX	6-FAM
Modification of 3' probe	Iowa Black FQ	Iowa Black FQ

### Statistical analysis

Initially, normality tests (Shapiro–Wilk, Anderson–Darling, D'Agostino–Pearson, and Kolmogorov–Smirnov) were performed, and the dPCR data were found to be non-normally distributed. The nonparametric Kruskal–Wallis test was then applied to compare the amounts of microfilaria DNA from *D. immitis* and *Wolbachia* DNA on each day of treatment, and the Spearman test was applied to correlate the counts of microfilaria DNA from *D. immitis* and *Wolbachia* DNA at each timepoint.

To account for non-normality and intrinsic variance in the study, a spatial pairwise comparison was used to assess the proportions of *Wolbachia* vs. *D. immitis* microfilariae. The proportions of *Wolbachia* DNA, quantified as copy number per µL (cp/µl), and *D. immitis* DNA cp/µl were calculated for each timepoint using the equation, proportion = *Wolbachia* DNA cp/µl ÷ *D. immitis* DNA cp/µl. The arcsine transformation was applied to these proportions. A mixed model was used for the pairwise comparisons across all data and across each dog's origin (Rio, Angra, Marica, and São Pedro). Tukey’s adjustment method for multiple pairwise comparisons was used to control the overall significance level.

## Results

### Dogs

A total of 228 animals were sampled to identify qualified dogs. Thirty-two dogs were identified as fitting the criteria for inclusion in the study. Of those dogs, 31 microfilaremic dogs completed the doxycycline treatment. One dog was removed from the study due to vomiting episodes that started 24 h after the first doxycycline dose. The dog did not respond to any antiemetic therapy for 48 h. One additional amicrofilaremic *D. immitis* antigen-positive dog (dog number 25) was sampled according to the protocol, treated with doxycycline and had its samples shipped to LVCP/UFPR. However, this dog, number 25, was ultimately removed from the study.

### Microfilaria counts (Knott’s test results)

Of the 31 microfilaremic dogs that completed the study, 16 were male, and 15 were female. The average age was 5.8 with a standard deviation of ± 2.8 years. Most dogs were over 6 years (48.4%; 15/31), followed by those 2–4 years (22.6%; 7/31), 4–6 years (16.1%; 5/31), and 1–2 years (12.9%; 4/31). Most dogs completing the protocol were from Maricá (41.9%; 13/31), followed by Angra, Rio, and São Pedro, each at 19.3% (6/31). Microfilaria counts ranged from 390 to 8790 on day 0, from 10 to 28940 on day 60, and from 0 to 5380 on day 90. Only 4 of the 31 dogs were considered amicrofilaremic on the Knott’s test by day 90 (Table 2).

**Table 2:** Microfilaria concentration (mf/ml) of *D. immitis* before treatment with doxycycline (D0), 60 (D60), and 90 (D90) days after the first dose of doxycycline from dogs from Rio de Janeiro, Brazil

Dog	D0	D60	D90	Dog	D0	D60	D90
1	1770	520	240	17	5290	150	10
2	2750	390	20	18	940	1300	190
3	3180	2490	990	19	1040	140	0
4	8790	2100	510	20	720	160	270
5	550	20	0	21	1880	90	130
6	890	100	470	22	1780	3830	300
7	2790	1400	100	23	5660	3520	160
8	900	400	340	24	1720	7280	950
9	480	1620	70	26	7940	10	80
10	6150	28,940	360	27	2300	1140	180
11	2900	1180	560	28	690	640	140
12	4300	200	380	29	400	210	40
13	450	1220	20	30	1950	180	0
14	580	400	200	31	8600	18,100	5380
15	2750	300	570	32	3840	300	0
16	390	300	10				

### dPCR analysis

Only one of the 31 dogs completing the study had no evidence of microfilariae on day 90 by dPCR analysis. By day 60, 13 dogs had no dPCR evidence of *Wolbachia*; by day 90, 25 dogs were cleared of *Wolbachia* by dPCR (Table 3).

**Table 3:** Individual dog microfilariae and *Wolbachia* dPCR results (copy number per µl) on days 0, 60, and 90 from the 31 microfilaremic dogs from Rio de Janeiro, Brazil

Dog origin	Dog ID	*Dirofilaria immitis* (cp/µl)			*Wolbachia* dPCR (cp/µl)		
		D0	D60	D90	D0	D60	D90
Angra	2	328.4	199.1	5.761	7.600	0	0
Angra	12	426.1	221.0	42.55	9.660	0.277	0
Angra	15	971.6	233.2	41.11	14.710	0	0
Angra	21	237.7	19.72	23.53	4.217	0	0
Angra	30	88.87	25.96	0.21	1.525	0.027	0
Angra	32	423.9	36.28	0.323	8.463	0	0
Maricá	4	7028.1	324.5	35.79	135.000	0	0
Maricá	6	1431.5	247.5	117.5	30.610	0.055	0
Maricá	7	208.2	23.73	1.182	3.738	0	0
Maricá	9	256.9	164.8	10.88	4.473	0,11	0
Maricá	10	2221.7	1072.6	38.8	53,210	0.541	0
Maricá	11	349.6	129.8	13.27	7.243	0.055	0
Maricá	13	758.2	334.8	0.864	16.920	0.11	0
Maricá	20	516.8	21.77	8.403	9.493	0	0
Maricá	24	534.0	183.9	20.78	9.527	0.055	0
Maricá	26	520.1	9.014	6.85	10.460	0	0
Maricá	28	227.45	146.2	26.61	4.020	0.054	0.189
Maricá	29	90.3	24.02	27.525	2.208	0	0
Maricá	31	6039.1	1220.35	85.15	105.400	0.711	0.082
Rio	1	322.1	323.6	67.44	7.548	0.277	0
Rio	3	994.4	853.1	655.0	19.290	0.104	0.053
Rio	8	1183.7	224.2	49.83	17.970	0.055	0.054
Rio	17	286.5	27.09	6.994	3.358	0	0
Rio	19	584.4	87.41	0.704	12.780	0.055	0
Rio	27	418.0	196.4	185.4	7.251	0.054	0.054
S. Pedro	5	268.0	17.35	0.272	3.991	0	0
S. Pedro	14	311.8	29.75	6.945	4.566	0.055	0
S. Pedro	16	504.2	13.38	0	6.377	0	0
S. Pedro	18	539.6	47.78	9.443	12.150	0	0
S. Pedro	22	1632.6	472.4	234.9	30.130	0.276	1.313
S. Pedro	23	2174.2	303.7	27.58	40.200	0.377	0

### Microfilaria and *Wolbachia* DNA quantification

For all data in Table 4, the comparisons between day 60 and day 90 and day 0 were statistically significant (*p* < 0.0001). Still, the comparison between day 60 and day 90 was not statistically significant (*p *= 0.3127) at α = 0.05. This conclusion is also consistent in the analyses for each region (Table 4). Thirteen dogs were dPCR negative for *Wolbachia* DNA by day 60 (32 days after the last doxycycline dose), and 25 of the 31 dogs were negative on day 90. This left six dogs that remained positive for *Wolbachia* DNA on day 90, with quantities rising from day 60 to day 90 in 2 of the 6, staying the same in 2, and falling in 2. However, *Wolbachia* DNA persisted at measurable levels in all six dogs.

**Table 4:** Data of arcsine-transformed proportion of *Wolbachia* DNA/*D. immitis* microfilariae DNA from dogs from Rio de Janeiro, Brazil

All Dogs	Variable	LSM*	Comparison	*p* value
	Arsin_Prop_Day0	0.1373	Arsin_Prop_Day0 vs. Arsin_Prop_Day60	< .0001
	Arsin_Prop_Day60	0.01388	Arsin_Prop_Day0 vs. Arsin_Prop_Day90	< .0001
	Arsin_Prop_Day90	0.00799	Arsin_Prop_Day60 vs. Arsin_Prop_Day90	0.3127
Rio Dogs	Arsin_Prop_Day0	0.1343	Arsin_Prop_Day0 vs. Arsin_Prop_Day60	< .0001
	Arsin_Prop_Day60	0.01627	Arsin_Prop_Day0 vs. Arsin_Prop_Day90	< .0001
	Arsin_Prop_Day90	0.009831	Arsin_Prop_Day60 vs. Arsin_Prop_Day90	0.6995
Angra Dogs	Arsin_Prop_Day0	0.139	Arsin_Prop_Day0 vs. Arsin_Prop_Day60	< .0001
	Arsin_Prop_Day60	0.01128	Arsin_Prop_Day0 vs. Arsin_Prop_Day90	< .0001
	Arsin_Prop_Day90	-1.82E-17	Arsin_Prop_Day60 vs. Arsin_Prop_Day90	0.2718
Marica Dogs	Arsin_Prop_Day0	0.1413	Arsin_Prop_Day0 vs. Arsin_Prop_Day60	< .0001
	Arsin_Prop_Day60	0.01251	Arsin_Prop_Day0 vs. Arsin_Prop_Day90	< .0001
	Arsin_Prop_Day90	0.008878	Arsin_Prop_Day60 vs. Arsin_Prop_Day90	0.8347
S. Pedro Dogs	Arsin_Prop_Day0	0.1299	Arsin_Prop_Day0 vs. Arsin_Prop_Day60	< .0001
	Arsin_Prop_Day60	0.01707	Arsin_Prop_Day0 vs. Arsin_Prop_Day90	< .0001
	Arsin_Prop_Day90	0.01306	Arsin_Prop_Day60 vs. Arsin_Prop_Day90	0.9477

^*^ LSM = Least square means

## Discussion

The proportional analysis between *Wolbachia* DNA and *D. immitis* DNA may provide an important perspective for interpreting the effect of doxycycline in *D. immitis-*infected dogs. Considering that the absolute number of circulating microfilariae varied substantially among dogs and across sampling times and that Knott’s modified mf counting does not completely eliminate subjectivity, evaluating the proportion of *Wolbachia* DNA to *D. immitis* DNA enabled a more objective, alternative assessment of the bacterial and parasite burdens in the blood. The comparison between day 0 and both post-treatment timepoints demonstrated a highly significant effect (*p* < 0.0001), indicating that doxycycline substantially reduced the relative abundance of *Wolbachia*. No statistically significant difference in *Wolbachia* DNA was observed between days 60 and 90 (*p* = 0.3127). This apparent stabilization of the *Wolbachia* amplification signal may reflect the persistence of low levels of bacterial DNA within the parasite population rather than continued bacterial clearance. Such persistence may be related to factors such as heterogeneous responses of *Wolbachia* populations to antibiotic exposure [20] and the presence of high adult parasite burdens in some dogs, but these hypotheses were not evaluated in the present study.

Several biological mechanisms may contribute to this phenomenon. Experimental studies have shown that *Wolbachia* can alter gene and protein expression in response to doxycycline exposure, including modulation of translation-related pathways, upregulation of metabolic and transport functions, and downregulation of growth-associated RNAs and surface proteins. These transcriptional and proteomic adjustments are consistent with physiological adaptation to antibiotic stress and may reduce bacterial susceptibility to the drug [20]. In addition, characteristics associated with antibiotic tolerance, such as low metabolic or dormant states analogous to bacterial persisters, have also been described in *Wolbachia* populations [20]. This potentially allows reversible survival during antibiotic exposure without requiring stable genetic resistance mechanisms—adopting a possibly transient detoxification mechanism.

Spatial heterogeneity in the distribution of *Wolbachia* within filarial tissues may represent an additional factor influencing bacterial persistence. One study in filarial models demonstrated that *Wolbachia* are often organized into localized clusters within specific parasite tissues rather than uniformly distributed throughout the host [23]. Dense bacterial clusters observed in reproductive tissues of *Brugia* spp., for example, have been shown to persist following antibiotic treatment and have been proposed as protected niches that allow bacterial survival and subsequent rebound after therapy [24]. Such spatial organization may generate bacterial subpopulations exposed to different antibiotic concentrations or physiological conditions, leading to heterogeneous susceptibility within the population and potentially different levels of drug efficacy.

An additional observation emerging from the present data is the persistence of circulating microfilariae in a substantial proportion of dogs despite the marked reduction in detectable *Wolbachia* DNA. While doxycycline treatment significantly reduced bacterial burden, most animals remained microfilaremic during data collection. This finding reinforces that circulating microfilariae may persist even when the associated *Wolbachia* burden is markedly reduced or below the detection threshold. Such observations are consistent with previous studies demonstrating that doxycycline primarily affects embryogenesis and long-term parasite viability rather than immediately eliminating circulating microfilariae [25, 26].

Another important consideration relates to the molecular method used in this study. Digital PCR provides greater analytical sensitivity and lower detection thresholds than quantitative PCR [27–30], which has been used in most previous studies evaluating *Wolbachia* elimination in *D. immitis*. Consequently, the detection of small amounts of residual *Wolbachia* DNA in some animals may reflect the dPCR assay's increased sensitivity rather than a lack of treatment effect, even when accounting for DNA from dead organisms.

It is important to consider that the treatment protocol used in the present study differs from that used in many previous investigations evaluating the effect of doxycycline on *Wolbachia* in *D. immitis*. In several experimental and clinical studies, doxycycline has been administered alongside a macrocyclic lactone [18, 31], whereas in the present study, dogs received doxycycline alone throughout the observation period. The concurrent use of a macrocyclic lactone can influence microfilarial dynamics and may also contribute to the overall reduction of *Wolbachia* within the parasite population [31]. Because of this difference in treatment protocols, direct comparisons between studies should be interpreted with caution. Therefore, the persistence of detectable *Wolbachia* DNA in a small number of dogs in the present study reflects, at least in part, the absence of this combined therapeutic approach rather than a fundamentally different biological response.

As expected, despite the relatively small sample size, the high-dose doxycycline regimes greatly reduced microfilarial burdens through the 90-day observation period. However, complete elimination of *Wolbachia* DNA within 30 days following completion of a doxycycline treatment protocol, as previously reported [14], was not achieved in some animals by the 90th day. This discrepancy may again be attributable to our not using a macrocyclic lactone, as was done in the study cited in reference [14]. This study demonstrated that *Wolbachia* DNA can be persistently detected in some animals for up to at least 90 days after doxycycline-only treatment. As such, doxycycline-only treatment is not recommended for treating heartworm infections in dogs.

Although these findings provide insight into *Wolbachia* dynamics following doxycycline treatment, several aspects of the study design should be considered when interpreting the data. The number of dogs included in the study was relatively limited, and no untreated control group was available for comparison. In addition, dogs did not receive macrocyclic lactones during the study period, unlike many previous investigations evaluating the effects of doxycycline on *Wolbachia* [14, 18, 31]. Another consideration is that the molecular detection of *Wolbachia* DNA does not necessarily indicate the presence of viable bacteria, particularly when highly sensitive techniques such as dPCR are employed [28, 30]. Finally, host-related factors such as prior antibiotic exposure, immune status, or parasite burden may also contribute to variation in *Wolbachia* clearance among individual animals [32]. Differences in drug absorption and pharmacokinetics between dogs could also influence doxycycline exposure and may partly explain variation in *Wolbachia* clearance [33].

The best control of heartworm populations would be achieved through a multivalent approach, treating adult infections with melarsomine and preventing new infections with insect repellents and macrocyclic lactones, rather than relying on a single modality. For heartworm disease, the strategy would be to use macrocyclic lactones in combination with vector-control compounds, such as repellents or isoxazolines [34–36]. For treating adult heartworm infections, melarsomine, an effective adulticidal arsenical drug, is typically used, offering a completely different mode of action against the parasite. Unfortunately, melarsomine is currently unavailable for routine use in Brazil and many countries, where heartworms are endemic [37, 38]. It would be advisable for regulatory authorities and pharmaceutical companies to consider reintroducing melarsomine in Brazil and other Latin American countries.

## Conclusions

This study demonstrates that *Wolbachia* DNA may persist in some dogs for 90 days after doxycycline-only therapy. This is likely due to the absence of a macrocyclic lactone in the treatment protocol or to higher adult worm burden in some dogs, but further studies should investigate whether this is related to resistance to doxycycline in certain *Wolbachia* subpopulations or to other factors.

## Supplementary Information

Supplementary Material 1.

## Data Availability

All data are available in the manuscript and accompanying supplementary material.
