# Supplementary material for: Persistence of Wolbachia DNA in Dirofilaria immitis-infected canines after doxycycline-only treatment in Rio de Janeiro State, Brazil
Source: Parasit Vectors. 2026 Sep 1;19:480. doi: 10.1186/s13071-026-07621-9 (PMC13741592; doi:10.1186/s13071-026-07621-9)
Supplement: Supplementary file 1 [file 13071_2026_7621_MOESM1_ESM.docx]

**Supplementary Material**

**Quantification of *D. immitis* and *Wolbachia* by dPCR**

The follow-up of microfilaremia can be performed by the modified Knott’s test [S1], while the quantification of Wolbachia falls to in-depth molecular methods. Among these methods, the most popular is real-time PCR (qPCR) [S2]. Although qPCR is widely used for the qualitative and quantitative analysis of a few pathogens, its use has been progressively replaced using digital PCR (dPCR). Similarly to qPCR, in dPCR, the quantification is also based on the intensity of the fluorescence captured during the amplification of the target, using the same primers and probes applied in qPCR. The difference between these techniques lies in the preparation and partitioning of the sample, which gives dPCR more sensitive and accurate results than qPCR. In addition, dPCR provides greater reproducibility and repeatability of the final data [S3,S4]. Despite the advantages of dPCR over qPCR, no studies have been found in the literature that apply this technique in the quantification of *D. immitis* and its *Wolbachia* endosymbiont.

**Digital Polymerase Chain Reaction (dPCR)**

Species-specific primers and IDT probes (Iowa, USA) [S2] were used for dPCR (Table 1). The samples were standardized by first testing the primers in a conventional PCR. Subsequently, variations in the concentration of probes and primers, amount of initial DNA, and temperature in the dPCR reactions were tested. After standardization, duplex dPCR reactions were performed with a final volume of 40 μL. The reaction was set up under the following conditions: 20 ng of DNA; 1 X Probe PCR Master Mix from Qiagen (Hilden, Germany), 0.8 μM from each primer for *Wolbachia*, 0.4 μM probe for *Wolbachia*, 0.6 μM of each primer for *D. immitis*; 0.3 μM probe for *D. immitis*; 0.2 IU of EcoRI from Thermo Scientific (Massachusetts, USA). The total volume of the reaction was pipetted into the well of Qiagen's Nanoplate 26K 24-well plate (Hilden, Germany). The reaction was held on Qiagen's QIAcuity One equipment (Hilden, Germany) under the following temperature conditions: 1 cycle of 95 °C for 2 min, 55 cycles of 95 °C for 15 sec, 58 °C for 15 sec and 72 °C for 15 sec. The exposure time was 500 ms, and the gain was 6. A negative control without DNA (NTC) was inserted in all plates. The fluorescence cut-off line (threshold) was manually determined for all samples, following the established methodology for the equipment.

**Supplementary Data Table 1.** Primers and probes were used to run dPCR for *Dirofilaria immitis* and *Wolbachia*.

|  | *D. immitis* (gene Cox-1) | *Wolbachia* (gene Ftsz) |
| --- | --- | --- |
| Forward 5’ | CATCCTGAGGTTTATGTTATTATTTT | AAGCCATTTGGCTTCGAAGGTG |
| Reverse 3’ | CTGTATACATATGATGACCCCA | AAACAAGTTTTGATTTGGAATAACAAT |
| Probe | CGGTGTTTGGGATTGTTAGTG | CGTATTGCAGAGCTCGGATTA |
| Modification of 5' probe | HEX | 6-FAM |
| Modification of 3' probe | Iowa Black FQ | Iowa Black FQ |

**Standardization of dPCR**

After DNA extraction from the sample of a patient known to be infected with *D. immitis* (presence of microfilariae in the modified Knott’s test and antigens in the rapid antigen test), a conventional PCR was performed to test the primers. As shown in Supplementary Figure S1, PCR using primers for detecting *D. immitis* and *Wolbachia* resulted in a single band of the expected size (166 and 111 bp, respectively), with no nonspecific bands or the formation of primer dimers.

**MM**

**1**

**2**

100pb

500pb

**Supplementary Figure 1.** Conventional PCR used to test primers for *Dirofilaria immitis* and *Wolbachia*. MM Line: Molecular weight marker (100 bp). Line 1: PCR for *Wolbachia* amplification (red arrow: 111 bp band). Line 2: PCR for amplification of *Dirofilaria immitis* (purple arrow: 166 bp band).

Since the primers did not show problems in conventional PCR, dPCR standardization tests were performed. To verify the proper functioning of the probes and ensure that there was no interference in detecting fluorescence, a test was performed using only the primers and probe specific for *Wolbachia*. The same conditions were repeated using the primers and probes specific for each agent in the same reaction. Supplementary Figure S2 illustrates one of the samples tested initially with only the probe for *Wolbachia* (A) and then with the duplex for *Wolbachia* (B) and *D. immitis* (C). The assays with this sample returned the values of 7,100 copies/μl of Wolbachia when the singleplex was tested. When the duplex was tested, we obtained 7,017 copies/μl of *Wolbachia* and 235.7 copies/μL of *D. immitis*.

In this test, it was possible to observe that the fluorescence of the specific probe for *D. immitis* did not interfere with the fluorescence reading of the specific probe for *Wolbachia*, and it was possible to use both probes in the same reaction (duplex dPCR). In addition, it was possible to identify that the *Wolbachia* amplification reaction showed a clear separation between positive and negative partitions, which is desirable for this technique. However, in the reaction for amplification of *D. immitis* (Supplementary Figure S2C), intermediate fluorescence partitions (rain effect) were observed, complicating the interpretation of whether such partitions were positive or negative. To minimize this problem, new standardization tests were performed, changing the amount of initial DNA, the primers' concentration, the probes' concentration, and the girdling temperature.

A

B

C

**Supplementary Figure S2.** 1D scatter plot. Each point equals a partition. The blue dots above the red line (threshold) correspond to the positive partitions, and the ones below the line (gray dots) correspond to the negative partitions for the target. (A) Singleplex dPCR for *Wolbachia*; (B. and C) duplex dPCR for *Wolbachia* (B) and *D. immitis* (C).

Probe and primer concentrations for *D. immitis* of 0.3 and 0.6 μM were tested: 0.4 and 0.8 μM and 0.6 and 1.2 μM. Reactions with 160, 80, 40, and 20 ng of DNA were tested at 60 and 58°C annealing temperatures with 35 and 55 cycles. Although the rain effect was still present, the lower concentrations of the probe and primers (0.3 and 0.6 μM, respectively) showed better results than the other concentrations (Figure S3).

A

B

C

**Supplementary Figure S3.** 1D scatter plot. Same sample and reactions with probe concentration and primers for *D. immitis* of 0.3 and 0.6 μM (A), 0.4 and 0.8 μM (B), and 0.6 and 1.2 μM (C).

Regarding the annealing temperature and the number of cycles, reducing the temperature from 60 to 58 °C and adding more cycles (55 cycles) led to a greater definition of positive partitions (Supplementary Figure S4).

A

B

**Extended Data Figure S4.** 1D scatter plot. dPCR for detection of *D. immitis*. Reactions with 40ng of DNA and probe and primer concentration at 0.3 and 0.6 μM, respectively. A. Annealing temperature of 60°C and 35 cycles. B. Annealing temperature of 58°C and 55 cycles.

However, a better result was obtained when different amounts of DNA were tested. Supplementary Figure S5 shows that with 20 ng of DNA, there was a better distinction between the positive and negative partitions for *D. immitis* without impeding the detection of *Wolbachia*.

**Supplementary Figure S5.** 1D scatter plot of dPCR for *D. immitis* with the different amounts of DNA. With 20 ng of DNA, there was a reduction in the rain effect, with a better separation of positive and negative partitions.

After the standardization tests, the reaction conditions were established: the amount of DNA of 20 ng, annealing temperature of 58 °C with 55 cycles. The probe and primer concentrations for *Wolbachia* were 0.4 and 0.8μM, and for D. immitis, they were 0.3 and 0.6μM. (Supplmentary Figure S6).

A

B

**Supplementary Figure S6.** 1D duplex dPCR scatter plot using the standardized conditions. (A) Result for *D. immitis*. (B) Result for *Wolbachia*.

A

B

**Supplementary Figure S7.** Quantification of *Dirofilaria immitis* (A) and *Wolbachia* (B) at D0, D+60, and D+90.

Supplementary Table S2 shows the copy number/μl of *D. immitis* and *Wolbachia* in the dPCR reaction before treatment with doxycycline (D0), 60 and 90 days after treatment (D60 and D90). Animal 25 was excluded from statistical analyses because it was not positive for *D. immitis* in both the dPCR and Knott’s test and even *Wolbachia* in the dPCR. The evaluation of the data showed a significant reduction (P < 0.001) in the amount of *D. immitis* and *Wolbachia* over the treatment (Supplementary Figure S7A and B).

**Supplementary Table S2.** Copy number/μl of *Dirofilaria immitis* and *Wolbachia* in the dPCR reaction before starting treatment with doxycycline (D0) and D60, and D90 after treatment.

|  |  | *Dirofilaria immitis* | | |  | *Wolbachia* | | |
| --- | --- | --- | --- | --- | --- | --- | --- | --- |
| DOG ORIGIN | DOG ID | D0 | D60 | D90 |  | D0 | D60 | D90 |
| Rio | 1 | 322.1 | 323.6 | 67.44 |  | 7.548 | 0.277 | 0 |
| Angra | 2 | 328.4 | 199.1 | 5.761 |  | 7.600 | 0 | 0 |
| Rio | 3 | 994.4 | 853.1 | 655 |  | 19.290 | 0.104 | 0.053 |
| Maricá | 4 | 7028.1 | 324.5 | 35.79 |  | 135.000 | 0 | 0 |
| S. Pedro | 5 | 268 | 17.35 | 0.272 |  | 3.991 | 0 | 0 |
| Maricá | 6 | 1431.5 | 247.5 | 117.5 |  | 30.610 | 0.055 | 0 |
| Maricá | 7 | 208.2 | 23.73 | 1.182 |  | 3.738 | 0 | 0 |
| Rio | 8 | 1183.7 | 224.2 | 49.83 |  | 17.970 | 0.055 | 0.054 |
| Maricá | 9 | 256.9 | 164.8 | 10.88 |  | 4.473 | 0.11 | 0 |
| Maricá | 10 | 2221.7 | 1072.6 | 38.8 |  | 53.210 | 0.541 | 0 |
| Maricá | 11 | 349.6 | 129.8 | 13.27 |  | 7.243 | 0.055 | 0 |
| Angra | 12 | 426.1 | 221 | 42.55 |  | 9.660 | 0.277 | 0 |
| Maricá | 13 | 758.2 | 334.8 | 0.864 |  | 16.920 | 0.11 | 0 |
| S. Pedro | 14 | 311.8 | 29.75 | 6.945 |  | 4.566 | 0.055 | 0 |
| Angra | 15 | 971.6 | 233.2 | 41.11 |  | 14.710 | 0 | 0 |
| S. Pedro | 16 | 504.2 | 13.38 | 0 |  | 6.377 | 0 | 0 |
| Rio | 17 | 286.5 | 27.09 | 6.994 |  | 3.358 | 0 | 0 |
| S. Pedro | 18 | 539.6 | 47.78 | 9.443 |  | 12.150 | 0 | 0 |
| Rio | 19 | 584.4 | 87.41 | 0.704 |  | 12.780 | 0.055 | 0 |
| Maricá | 20 | 516.8 | 21.77 | 8.403 |  | 9.493 | 0 | 0 |
| Angra | 21 | 237.7 | 19.72 | 23.53 |  | 4.217 | 0 | 0 |
| S. Pedro | 22 | 1632.6 | 472.4 | 234.9 |  | 30.130 | 0.276 | 1.313 |
| S. Pedro | 23 | 2174.2 | 303.7 | 27.58 |  | 40.200 | 0.377 | 0 |
| Maricá | 24 | 534 | 183.9 | 20.78 |  | 9.527 | 0.055 | 0 |
| Maricá | 25* | 0 | 0 | 0 |  | 0 | 0 | 0 |
| Maricá | 26 | 520.1 | 9.014 | 6.85 |  | 10.460 | 0 | 0 |
| Rio | 27 | 418 | 196.4 | 185.4 |  | 7.251 | 0.054 | 0.054 |
| Maricá | 28 | 227.45 | 146.2 | 26.61 |  | 4.020 | 0.054 | 0.189 |
| Maricá | 29 | 90.3 | 24.02 | 27.525 |  | 2.208 | 0 | 0 |
| Angra | 30 | 88.87 | 25.96 | 0.21 |  | 1.525 | 0.027 | 0 |
| Maricá | 31 | 6039.1 | 1220.35 | 85.15 |  | 105.400 | 0.711 | 0.082 |
| Angra | 32 | 423.9 | 36.28 | 0.323 |  | 8.463 | 0 | 0 |

Rio – Rio de Janeiro Municipality; Angra – Angra dos Reis Municipality; Maricá Municipality; S. Pedro – São Pedro da Aldeia Municipality

* Control dog

At day 60, after the first Doxy dose, 42% (13/31) of the dogs had no *Wolbachia* DNA detected. The comparison of the relation no of *D. immitis* DNA copies: no of *Wolbachia* DNA copies (Di:Wo relation) of D0 with D+60 showed that the 18 dogs presenting *Wolbachia* DNA at D+60 when compared to the 31 dogs included at D0 showed that 58% had a higher Di:Wo relation, demonstrating that *Wolbachia* DNA load was lower at D+60. This suggests that there could be surviving microfilariae harboring no *Wolbachia*, surviving microfilariae harboring less *Wolbachia*, or that adult worms are producing microfilariae with reduced *Wolbachia* load (Supplementary Table S3).

At day 90, after the first Doxy dose, 81 % (25/31) of the dogs had no *Wolbachia* DNA detected. The comparison of the relation no of D. immitis DNA copies: no of Wolbachia DNA copies showed that among the six dogs presenting *Wolbachia* DNA at day 90, only 1 had a higher relation (less *Wolbachia* DNA). In contrast, 5 presented a lowered relation (increased Wolbachia DNA). This shift could suggest that after Doxy pressure was withdrawn, the microfilariae *Wolbachia* rebound or that the adult worms began to produce microfilariae harboring higher *Wolbachia* load.

**Supplementary Table S3.** The mean of copies /μl of *Dirofilaria immitis* and *Wolbachia* DNA and relation of the mean of copies of *D. immitis*/means of copies of *Wolbachia* before starting treatment with doxycycline (D0), on D+60 and D+90.

| **Day** | **N** | **Di** | **N** | **Wolb** | **N** | **Di:W** |  | **Di:W** |  | **Di:W** |
| --- | --- | --- | --- | --- | --- | --- | --- | --- | --- | --- |
|  |  | $\overset{¯}{x}$ |  | $\overset{¯}{x}$ |  | $\overset{¯}{x}$ |  | Increased |  | Decreased |
| D0 | 31 | 1028.3232 | 31 | 19.48670968 | 31 | 54.70488125 |  | - |  | - |
|  |  |  |  |  |  |  |  | D0 - D60 |  | D0 - D60 |
| D60 | 31 | 233.367871 | 31 | 0.1047411935 | 18 | 2481.2444 |  | 18 dogs |  | 0 dogs |
|  |  |  |  |  |  |  |  | D60 - D90 |  | D60 - D90 |
| D90 | 31 | 56.503097 | 31 | 0.056274194 | 6 | 3012.181116 |  | 1 dog |  | 5 dogs |

Supplementary Figure S8 shows the oscillation in the quantification of *D. immitis* and *Wolbachia* among D0, D60, and D90, and a positive (D0 and D60) correlation between *D. immitis* was observed. Spearman correlation measures the strength of a relationship between two variables. It is a non-parametric measure, meaning it does not assume any specific distribution for the variables. The value ranges from -1 to +1. A value of +1 indicates a perfect positive monotonic relationship, and -1 indicates an ideal negative monotonic relationship. The correlation of *D. immitis* at D0 and D60 was 0.714 (moderate to strong).

|  | Series1 | Series2 | Series3 | Series4 | Series5 | Series6 | Series7 | Series8 | Series9 | Series10 | Series11 | Series12 | Series13 | Series14 | Series15 | Series16 | Series17 | Series18 | Series19 | Series20 |
| --- | --- | --- | --- | --- | --- | --- | --- | --- | --- | --- | --- | --- | --- | --- | --- | --- | --- | --- | --- | --- |
| 1 | 7.548 | 7.6 | 19.29 | 135 | 3.991 | 30.61 | 3.738 | 17.97 | 4.473 | 53.21 | 7.243 | 9.66 | 16.92 | 4.566 | 14.71 | 6.377 | 3.358 | 12.15 | 12.78 | 9.493 |
| 2 | 0.277 | 0 | 0.104 | 0 | 0 | 0.055 | 0 | 0.055 | 0.11 | 0.541 | 0.055 | 0.277 | 0.11 | 0.055 | 0 | 0 | 0 | 0 | 0.055 | 0 |
| 3 | 0 | 0 | 0.053 | 0 | 0 | 0 | 0 | 0.054 | 0 | 0 | 0 | 0 | 0 | 0 | 0 | 0 | 0 | 0 | 0 | 0 |

|  | Series1 | Series2 | Series3 | Series4 | Series5 | Series6 | Series7 | Series8 | Series9 | Series10 | Series11 | Series12 | Series13 | Series14 | Series15 | Series16 | Series17 | Series18 | Series19 | Series20 |
| --- | --- | --- | --- | --- | --- | --- | --- | --- | --- | --- | --- | --- | --- | --- | --- | --- | --- | --- | --- | --- |
| 1 | 322.1 | 328.4 | 994.4 | 7028.1 | 268 | 1431.5 | 208.2 | 1183.7 | 256.9 | 2221.7 | 349.6 | 426.1 | 758.2 | 311.8 | 971.6 | 504.2 | 286.5 | 539.6 | 584.4 | 516.8 |
| 2 | 323.6 | 199.1 | 853.1 | 324.5 | 17.35 | 247.5 | 23.73 | 224.2 | 164.8 | 1072.6 | 129.8 | 221 | 334.8 | 29.75 | 233.2 | 13.38 | 27.09 | 47.78 | 87.41 | 21.77 |
| 3 | 67.44 | 5.761 | 655 | 35.79 | 0.272 | 117.5 | 1.182 | 49.83 | 10.88 | 38.8 | 13.27 | 42.55 | 0.864 | 6.945 | 41.11 | 0 | 6.994 | 9.443 | 0.704 | 8.403 |

A	B

**Supplementary Figure S8.** Oscillation in quantifying individual dog microfilariae of *Dirofilaria immitis* (A) and *Wolbachia* (B) for doxycycline treatment.

**REFERENCES:**

S1. Newton, W.L. and Wright, W.L. (1956). The occurrence of a dog filariid other than *Dirofilaria immitis* in the United States. Journal of Parasitology, 42(3), 246-258.

S2. Laidoudi, Y., Laidoudi, Y., Davoust, B., Davoust, B., Varloud, M., Niang, E. H. A., Niang, E. H. A., Fenollar, F., Fenollar, F., Mediannikov, O., & Mediannikov, O. (2020). Development of a multiplex qPCR-based approach for the diagnosis of *Dirofilaria immitis*, *D. repens* and *Acanthocheilonema reconditum*. Parasites and Vectors, 13(1), AR. <https://doi.org/10.1186/s13071-020-04185-0>

S3. Huggett, J. F., Foy, C. A., Benes, V., Emslie, K., Garson, J. A., Haynes, R., Hellemans, J., Kubista, M., Mueller, R. D., Nolan, T., Pfaffl, M. W., Shipley, G. L., Vandesompele, J., Wittwer, C. T., & Bustin, S. A. (2013). The digital MIQE guidelines: Minimum information for publication of quantitative digital PCR experiments. *Clinical Chemistry*, *59*(6), 892–902. <https://doi.org/10.1373/clinchem.2013.206375>

S4. Whale, A. S., De Spiegelaere, W., Trypsteen, W., Nour, A. A., Bae, Y. K., Benes, V., Burke, D., Cleveland, M., Corbisier, P., Devonshire, A. S., Dong, L., Drandi, D., Foy, C. A., Garson, J. A., He, H. J., Hellemans, J., Kubista, M., Lievens, A., Makrigiorgos, M. G., … Huggett, J. F. (2020). The Digital MIQE Guidelines Update: Minimum Information for Publication of Quantitative Digital PCR Experiments for 2020. *Clinical Chemistry*, *66*(8), 1012–1029. <https://doi.org/10.1093/clinchem/hvaa125>

**Appendix 1.** Consent form to include the dogs in the study (after all the inclusion criteria).

INFORMED CONSENT

“**Molecular Characterization of *Wolbachia* of *Dirofilaria immitis* from the state of Rio de Janeiro”**

Comissão de Ética no Uso de Animais (CEUA) of the Instituto de Veterinária of the Universidade Federal Rural do Rio de Janeiro

(CIAEP nº 02.0081.2019 / Phone CEUA/IV/UFRRJ: 55 21 2682-3051

**Principal Investigator**: Bruno Ricardo Soares Alberigi da Silva

**Study objectives**: To study the bacteria of the genus *Wolbachia* of *Dirofilaria immitis* from the state of Rio de Janeiro.

**Procedures**: All the procedures follow the ethical elements on animal care and use of the Conselho Nacional de Controle de Experimentação Animal (Animal Use National Council; CONCEA) and approved by the Comissão de Ética no Uso de Animais (Ethics Commission for the Use of Animals; CEUA) of the Veterinary Institute of the Universidade Federal Rural do Rio de Janeiro under the number CEUA Nº2886031121. The included animals will be examined by palpation, auscultation and rectal temperature. A blood sample will be drawn from the dog’s arm or neck. The blood samples will be used for the diagnostics of Heartworm (*D. immitis*) infection. All the blood test results will be available to you. We will call you by phone on the days 7, 15, 30, 45 and 75 after the first Doxycycline administration to evaluate your dog’s wellbeing.

During the clinical exam or blood sampling, animals may get stressed and present painting. In that case the animal will be immediately withdrawn from the study and will stay loose to calm down. If, after the sampling the vein remain bleeding, a local compression will be made and as soon as the bleeding stops, the area will be cleaned.

**TIMELINE:** Your animal will be examined three times (days 0, 60 and 90), during those visits a blood sample will be drawn.

**Advantages:** Your dogs will be thoroughly examined and will have the result of Heartworm infection. If infected, the study will start the needed treatment, if uninfected, the dog may start chemoprophylaxis.

**Clarification**: Your dog’s inclusion in the study is voluntary. If you decide to remove your dog from the study, you may do it at any time, without loss.

Your personal information will be confidential, only the study personal will have access to them.

- The responsible Veterinarian for your dog will be Dr. Bruno Alberigi, CRMV-RJ nº11324. Besides him, the study team will also be responsible for the dog’s welfare during the study.
- If needed, you may call the team using:

Emergency phone: (21) 971907977

Team leader: Bruno Alberigi	Address: Km 07, rural zone, BR-465, Seropédica - RJ

**Consent declaration**

I have been fully enlightened on all the procedures of this study and it’s risks and advantages to my dog (s). I have also been informed that I can withdraw my animal (s) from the study at any time. By signing this CONSENT DECLARATION, I authorize the inclusion of my identified animal (s) in the study.

**Animal:**

Code:_________Name:___________________Breed:____________________________Sex:________ Age:_________

**Owner**

Name:______________________________________________ Phone:_______________________________

E-mail:______________________________________________

Address: _______________________________________Neighborhood:___________ City:_________ State:________

I am aware and I agree to make my personal information available for the study in which my dog (s) is taking part. My personal data will only be available for the research team, for the study control, not being made available for any other purpose. I declare to be aware that, to exercise my rights contained in the applicable legislation in relation to the protection of my personal data, I must send an e-mail to [alberigi@ufrrj.br](mailto:alberigi@ufrrj.bri) informing which right I want to exercise and aware that other information may be needed for such purpose.

Two copies of this document are signed. One stays with me and the other with the investigator.

__________________, _______/_______/______

City	dd	mm	yyyy

______________________________	_______________________________________________

Owner – Name and signature	Investigator – Name and signature

**Appendix 2.** Data capture form.

**Appendix 3**

**Figure 11.** 2D scatter plot after dPCR analysis. On the Y-axis is the fluorescence for *D. immitis*, and on the X-axis for *Wolbachia*. In gray, the negative partitions for both agents are represented. Yellow shows the positive partitions for *D. immitis* and the negative partitions for *Wolbachia*. In light blue are the negative partitions for *D. immitis* and positive for *Wolbachia*. The negative partitions for both agents are in dark blue. A - D: Each image refers to the analysis of a single nanoplate (4 plates in total).

A

B

C

D
